# Ergot Alkaloids in Wheat and Rye Derived Products in Italy

**DOI:** 10.3390/foods8050150

**Published:** 2019-05-01

**Authors:** Francesca Debegnach, Simona Patriarca, Carlo Brera, Emanuela Gregori, Elisa Sonego, Gabriele Moracci, Barbara De Santis

**Affiliations:** Reparto di Sicurezza Chimica degli Alimenti—Istituto Superiore di Sanità, 00161 Rome, Italy; francesca.debegnach@iss.it (F.D.); simona.patriarca88@gmail.com (S.P.); carlo.brera@iss.it (C.B.); emanuela.gregori@iss.it (E.G.); elison.mail@gmail.com (E.S.); gabriele.moracci@iss.it (G.M.)

**Keywords:** mycotoxins, ergot alkaloids, wheat, rye, UHPLC-MS/MS

## Abstract

Genus *Claviceps* is a plant pathogen able to produce a group of toxins, ergot alkaloids (EAs), whose effects have been known since the Middle Ages (ergotism). *Claviceps purpurea* is the most important representative specie, known to infect more than 400 monocotyledonous plants including economically important cereal grains (e.g., rye, wheat, triticale). EAs are not regulated as such. Maximum limits are in the pipeline of the EU Commission while at present ergot sclerotia content is set by the Regulation (EC) No. 1881/2006 in unprocessed cereals (0.05% as a maximum). This study aimed to investigate the presence of the six principal EAs (ergometrine, ergosine, ergocornine, α-ergocryptine, ergotamine and ergocristine) and their relative epimers (-inine forms) in rye- and wheat-based products. Of the samples, 85% resulted positive for at least one of the EAs. Wheat bread was the product with the highest number of positivity (56%), followed by wheat flour (26%). Rye and wheat bread samples showed the highest values when the sum of the EAs was considered, and durum wheat bread was the more contaminated sample (1142.6 μg/kg). These results suggest that ongoing monitoring of EAs in food products is critical until maximum limits are set.

## 1. Introduction

Mycotoxins are fungal toxin metabolites produced by a wide array of fungi. Several species of *Aspergillus, Penicillium* and *Fusarium* genera are responsible for the production of the mycotoxins of most concern (e.g., aflatoxins, ochratoxins and fumonisins among others). However, the genus *Claviceps,* which is a plant pathogen, is able to produce a group of mycotoxins, ergot alkaloids (EAs), whose effects have been known since the Middle Age (ergotism) [1]. Among the several species of *Claviceps*, the most important is the *Claviceps purpurea,* which is known to infect more than 400 plant species of monocotyledonous plants, grasses and economically important cereal grains such as rye, wheat, triticale, barley, millet and oat [2]. The fungal ascospores are windborne; they land, attach and germinate on the pistil surface of the plant during anthesis, initiating host–pathogen interaction that leads to a sclerotia production [3]. Sclerotia are the fruiting structure that achieve maturation after five weeks of fungal infection and contain alkaloids [3]. The presence of sclerotia has a double effect, on one hand sclerotia have a negative implication reducing the quality of grasses and grains because of the presence of different classes of alkaloids, being considered undesirable substances for animal and human health; on the other hand, because of the pharmacological effects, EAs have been industrially produced and used as active pharmaceutical drugs in medical applications predominantly used in obstetrics at the beginning of the 20th century, and in the treatment of migraine headaches [2,4]. EAs toxicity properties are known and have long since been characterised, past episodes of *Claviceps purpurea*-associated intoxication have been registered worldwide but more recent outbreaks of human poisoning by grain crops contaminated with sclerotia in developing country countries indicate that ergotism is still a disease of public health importance [5].

In 2012, upon a European Commission request, the European Food Safety Authority (EFSA) delivered a scientific opinion EAs in food and feed [4] where the establishment of a group acute reference dose (ARfD) of 1 μg/kg body weight (bw) and a group tolerable daily intake (TDI) of 0.6 μg/kg bw per day for the sum of the EAs were reported. These health-based guidance values (HBGVs) are based on the conclusions of the Contaminant Panel that defined the vasoconstrictive effect on tail muscular atrophy in rats as the critical effect for hazard characterisation [4].

With the aim to protect human and animal health, European Union (EU) measures are set to keep contaminants at levels that are toxicologically acceptable with maximum limits at defined levels, which are reasonably achievable by following good agricultural, fishery and manufacturing practices and taking into account the risk related to the food consumption [6]. In the case of EAs it happened that the scarce information available on the occurrence and on the correlation between sclerotia presence and EAs content did not allow the setting of appropriate limits. Hence, the EU published a recommendation [7] to ask to the member states and professional stakeholder organizations to produce data on ergot alkaloids including occurrence data and specific information on the relationship between the presence of ergot sclerotia and the level of individual EAs in food and feed, and to report to EFSA their findings. Presently, EAs are not regulated as such, but ergot sclerotia content is set by the Commission Regulation (EC) No. 1881/2006 in unprocessed cereals (with the exception of corn and rice) used for humans [6], the amount of ergot in food being restricted to 0.05% at a maximum. In addition, rye ergot (*Claviceps purpurea)* in feed materials and compound feed containing unground cereals is also restricted to a maximum content of 1000 mg/kg relative to moisture content of 12% [8]. The setting of appropriate and achievable maximum levels is in the pipeline of the European Commission and should be based on the sum of 12 EAs (ergometrine, ergosine, ergocornine α-ergocryptine, ergotamine and ergocristine and reletive epimers, ergometrinine, ergosinine, ergocorninine, α-ergocryptinine, ergotaminine and ergocristinine).

Liquid chromatographic (LC) methods with fluorometric or mass spectrometric detector (FLD or MS/MS) are available for the analysis of EAs with limit of quantification/detection (LOQ/LOD) in agreement with those to be achieved for the individual epimers as requested in the framework of the proposed maximum limits, 10 µg/kg for cereal grains, 4 µg/kg for cereal milling products and 2 µg/kg for final cereal based consumer products. Ranges of LOQ/LOD between 0.01–0.5 μg/kg for HPLC-FLD or 0.15–2.78 μg/kg for LC-MS/MS have been achieved and reported in literature [9,10,11,12]. The chromatographic run length needed for the separation of the 12 EAs and sample preparations represent an analytical constrain for the setting of HPLC-FLD methods [13]. More easily, LC-MS/MS methods guarantee the determination of the 12, usually with positive mode electrospray ionization (ESI+). Since epimerization is hard to control during sample extraction and analysis, it is always the best option to analyse both -ine and -inine form and specify the EAs content as sum. Enzyme-linked immunosorbent assay (ELISA) techniques have been developed and represent an option for a rapid and low-priced screening of EAs in agricultural crops and grain flour. However, being less specific and less accurate than LC-FLD or LC-MS/MS methods, they are less attractive than dipstick type tests, which represent the preferable option for testing EAs presence in the field.

Occurrence data on EAs has increased in the last five to eight years and EFSA could deliver a risk assessment opinion thanks to the availability of analytical results derived from food samples collected between 2011 and 2016 in 15 different European countries. More than 50% of the data came from the Netherlands and around the 28% from Germany, Italy being very scarcely represented [14]. From a general overview of the EFSA opinion, the highest levels of EAs in food were found in rye and rye-containing commodities, in particular the “rye milling products” category showed an upper bound-lower bound (LB–UB) range of 198–239 μg/kg. Among processed food, the highest levels of EAs were found in “mixed wheat and rye bread and rolls” (33–82 μg/kg), “rye bread and rolls” and “rye flakes” with ranges of LB-UB of 29–67 μg/kg and 35–83 μg/kg, respectively [14].

The aim of this study was to make available an EAs survey on grain and grain-based products namely, wheat and rye flours and bread from the Italian market. An LC-MS/MS method for the determination of the six principal alkaloids (ergometrine, ergosine, ergocornine, α-ergocriptine, ergotamine and ergocristine) and the relative epimers, -inine forms (ergometrinine, ergosinine, ergocorninine, α-ergocryptinine, ergotaminine and ergocristinine) was used for the monitoring of wheat- and rye-based products, after performance characteristics verification.

## 2. Materials and Methods

### 2.1. Chemicals and Reagents

Chemicals and solvents used for sample preparation were ‘pro-analysis’ quality or better. Milli-Q (Merk Millipore S.p.a, Milano, Italy) water was used. LC-MS grade acetonitrile (ACN) and ammonium carbonate (NH_4_)_2_CO_3_ were purchased from Sigma-Aldrich (Milano, Italy). Bondesil (Agilent Technologies, Santa Clara, CA, USA) Primary Secondary Amine (PSA) was used.

The analytical reference standards of ergometrine, ergosine, ergocornine, α-ergocryptine, ergotamine, ergocristine, ergometrinine, ergosinine, ergocorninine, α-ergocryptinine, ergotaminine, ergocristinine were purchased as dry film from Biopure (Tulln, Austria).

A standard mix solution was prepared in 100% ACN as a mix of all the EAs under examination at a concentration of 0.5 μg/mL of each of the alkaloids. This solution was diluted in ACN at 10 ng/mL to obtain a standard working solution used for preparation of calibration curves. All standard solutions were stored at −20 °C in amber vials.

### 2.2. Samples

A total of 71 samples of flour and bread, all 100% wheat or rye, were collected. Following the FoodEx level 2 coding, “grain milling products” and “bread and rolls” were taken. In particular 20 flour samples (16 wheat and 4 rye milling products) and 51 bread samples (39 wheat and 12 rye bread and rolls) were available. Products were purchased in different retail markets as shelf products.

All samples were homogenized prior to analysis; flours were mixed before taking an aliquot whilst bread samples were stored frozen at −20 °C and grounded into a fine powder directly before analysis.

### 2.3. Sample Preparation

For the EAs extraction and purification, the procedure described by Krska [9] was followed with minor modifications. Briefly, 10 g of sample were extracted with 50 mL of ACN/ammonium carbonate buffer (200 mg/L, pH 8.9) 84/16 shaking for 30 min. The extract was filtered with filter paper. One millilitre of the extract was transferred in an amber vial containing 0.05 g of PSA and vortexed for 45 s. Finally, the sample was filtered through a polytetrafluoroethylene membrane filter (PTFE 0.22 μm) and submitted to analysis. During all steps of the procedure samples were kept away from light.

### 2.4. Sample Analysis

Chromatographic separation was performed using Ultra-High-Performance Liquid Chromatography (UHPLC) with Waters RP Acquity UHPLC BEH column (50 × 2.1 mm, 1.7 μm, Milford, MA, USA). The flow rate was set at 0.4 mL/min and the column temperature at 40 °C. The mobile phases composition was optimized from Mulder et al. [15]. The mobile phase A was 10 mM ammonium carbonate aqueous solution at pH 10 and mobile phase B was 100% ACN. The separation was obtained with a short chromatographic run with the following gradient: 10% B increased to 70% in 3.67 min, kept isocratic at 70% B until 3.73 min then returned to 10% B at 3.77 min and finally re-equilibrated the column at 10% B until 5 min. The injection was performed in a total loop with a volume of 10 µL.

The mass spectrometric analysis was carried out with a triple quadrupole, Quattro Premier XE (Waters Milford, MA, USA) in Multiple Reaction Monitoring (MRM) acquisition mode with an ElectroSpray Ionization (ESI) interface. The analysis was performed in positive ion-mode, the precursor and product ions selected for each EA [16], together with the optimized MS/MS parameters are reported in Table 1. For each EA under examination, the most intense transition was used as the quantifier ion, and a second transition was used as the qualifier ion.

EAs quantification was performed with the external standard method, for this purpose, a matrix matched calibration curve for each matrix under examination was prepared at each analytical session.

### 2.5. Method Performances

The method was verified to assess the suitability for the selected matrices. Blank samples of whole wheat flour, wheat bread, rye flour and rye bread were used to test the method performances. For each matrix apparent recovery (R_A_), extraction recovery (R_E_) and matrix effect, in terms of signal suppression/enhancement (SSE) were assessed by preparing three calibration curves set on 5 concentration levels in pure solvent, in spiked extract and spiked samples [17]. Apparent recoveries were in the range 67–135% and 68–145% for wheat and rye matrices respectively, with mean values of 103% ± 17% and 106% ± 20% for wheat and rye, respectively. Extraction efficiency resulted comparable for wheat 85% ± 11% and rye 85% ± 12%, respectively. The presence of the matrix resulted in a general enhancement with few exceptions, giving a mean value of 122% ± 22% and 123% ± 16% for wheat and rye, respectively. Precision was evaluated in terms of relative standard deviation (RSD), values obtained were under 25% for all the EA/matrix combination, with the exception of ergocryptinine (RSD = 26). Despite the absence of specific official reference, the obtained values were considered satisfactory under the criteria of Regulation (EC) 401/2006. Linearity was checked in the working range and the parameter *R*^2^ > 0.995 set was always satisfied. The LOQ was 2.5 μg/kg for all the selected EAs. The LOQ contamination level was included in the validation being the first point of the calibration curve. Identification criteria were set according to the European guidance document for mycotoxin [18], in particular the retention time and ion ratio (qualifier/quantifier) were set using values obtained from the injected pure solvent calibration curves.

The detailed performance characteristics of the method are reported in the Supplementary Material (Table S1).

The method was also tested in a proficiency test (PT) organized by the European Union Reference Laboratory for Mycotoxins (EURL-Mycotoxins). Performances obtained confirmed the suitability of the method.

## 3. Results and Discussion

**Figure 1:**
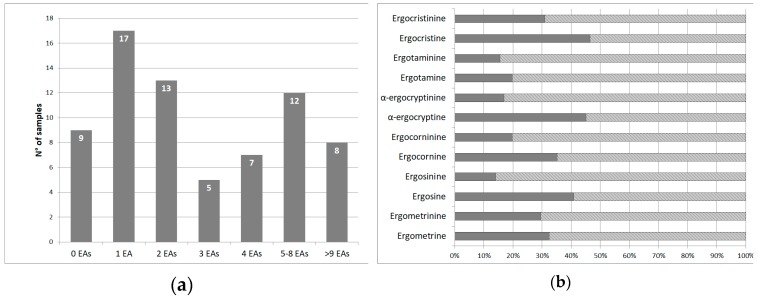
(**a**) Histograms representing the distribution of the number of EAs quantified per sample. (**b**) Percentage of samples resulted above or below the limit of quantification (LOQ) value for each EA, the percentage of positive samples being the solid grey portion of the histogram.

Of the 71 samples analysed, 62 samples (87%) were found to be contaminated with at least one of the 12 analysed EAs. In Figure 1a the number of EAs co-occurring in the analysed samples is reported; among the positive samples, 27% contained only one EA, while the eight samples (13%), four wheat-based and four rye-based, presented more than nine different EAs. The histograms of Figure 1b indicate the percentage of samples resulted above or below the LOQ value for each EA, the percentage of positive samples being the solid grey histogram.

The percentage of positive samples, the mean values (calculated with a medium bound approach), the standard deviation (s_r_) and the maximum contamination level for each of the analysed EAs are reported in Table 2.

**Figure 2:**
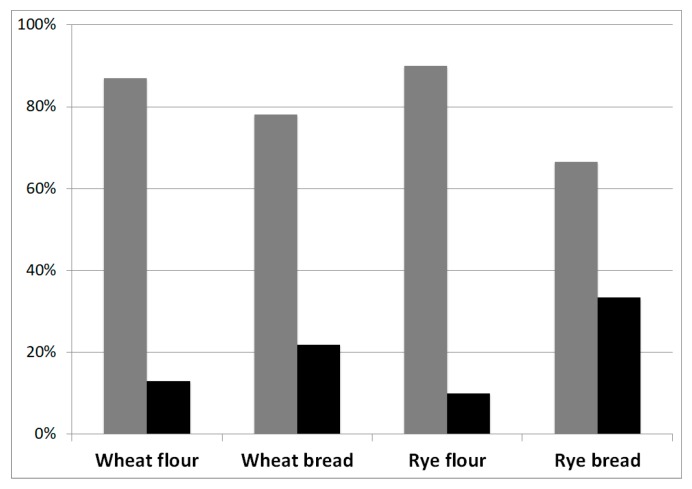
Histograms representing the percentage of -ine (grey histogram) and -inine (black histogram) content in each analysed food category.

Among the analysed samples, the most frequent EAs determined in the rye-based samples were ergocristine and ergocristinine in accordance with previous data reported in the literature [19,20], while in the wheat-based products, the most frequent EA was ergometrine together with its epimer ergometrinine. In general, the average concentration of the -ine forms was much higher than that of the -inine forms with a percentage (77% and 23%) being very close to that one found in the EFSA dataset (73% and 27%) [14]. A difference was observed between the -ine/-inine ratio in flour and bread samples (Figure 2). Indeed, the ratio between the average concentration of the epimeric forms shifts towards the -inine epimeric forms, probably due to the processing activities, like baking as already reported by Merkel et al. [21].

**Figure 3:**
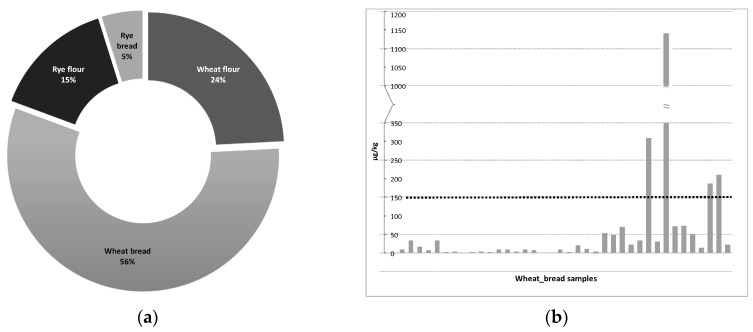
(**a**) Distribution (percentages) of the positive samples among the collected sample categories. (**b**) EAs contamination level in wheat bread samples, expressed as the sum of the 12 EAs analysed (µg/kg). The dotted line representing a possible EU maximum level for products intended for direct human consumption.

The distribution of the positive samples through the products is described in the Figure 3a, the pie chart shows that the most contaminated category was wheat bread. In the wheat bread samples, all the analysed EAs (-ine and -inine forms) were present with different co-occurrences. Of the samples, 56% were positive for ergocristine and the 46% for α-ergocryptine. The higher mean value was registered for the ergotamine (12.7 µg/kg), while the maximum value for a single EA was 314.3 µg/kg again for ergotamine. Ergosine and ergocristine were present in the 58% of rye bread samples but the contamination levels were lower than those recorded for the wheat bread samples.

The maximum legal limit at EU level for EAs is in the pipeline. The limit will refer to the sum of the 12 EAs (ergometrine, ergosine, ergocornine, α-ergocryptine, ergotamine and ergocristine and their epimers). EAs sum of rye-based products presented values in the range 2.6–61.3 µg/kg for flour and 2.9–188.6 µg/kg for bread, respectively. Wheat products presented values of the sum in the range 2.5–28.6 µg/kg for flour and 2.5–1142.6 µg/kg for bread, respectively. Due to these levels, which are considerably lower than a possible maximum limit of 150 μg/kg, there is a minimal risk for rye products and wheat flour. On the contrary, bread showed the 10% (*n* = 4) of the samples with values above 150 μg/kg. In Figure 3b, the sum of the 12 analysed EAs is reported for each wheat bread sample analysed, being the most contaminated category. From the histograms is possible to see that the majority of the samples are far below the foreseen legal limit, samples exceeding this value are all whole wheat bread. Also present in the wheat bread category, is the sample showing the highest EAs content when the sum of the 12 EAs is considered, with a contamination level of 1142.6 μg/kg. Because of the EAs content reduction during bakery [4], flour samples are expected to be more contaminated in comparison with bread samples. A possible explanation for the different scenario emerging form our results, may be the number of samples analysed per category, with the wheat bread being the most abundant (39 samples). Moreover, the whole wheat flour samples are three out of 16 (19%), while the collected whole wheat bread samples are 14 out of 39 (36%).

## 4. Conclusions

This study presents a survey on EAs of rye- and wheat-based products in Italy. Due to the dietary habits, wheat-based flour and bread were mostly represented (26%). The results of our study show generally low risk, especially for rye-based products (range for EAs sum 2.6–188.6 µg/kg) and wheat flour (range for the EAs sum 2.5–28.6 µg/kg). Wheat bread was the most critical food category (range for the EAs sum 2.5–1142.6 µg/kg) both for the presence of a very high value (1142.6 µg/kg) and for the number of products (*n* = 4) above a possible maximum limit of 150 μg/kg for cereal based products intended for direct human consumption. Ongoing monitoring of EAs in food products is recommended, at least for such a time until proper regulatory limits are set.

## Figures and Tables

**Table 1:** Optimized mass spectrometric (MS/MS) parameters for the selected ergot alkaloids (EAs).

Mycotoxin	Precursor Ion (m/z) ^1^	Product Ion (m/z) ^2^	Cone Voltage (V)	Collision Energy (V) ^2^
Ergometrine	326.3	223.1/208.0	20/20	25/30
Ergometrinine	326.3	208.0/223.1	20/20	30/25
Ergosine	348.2	223.1/208.1	20/20	25/30
Ergosinine	348.2	223.1/208.1	20/20	30/25
Ergocornine	562.2	222.9/304.9	20/20	35/25
Ergocorninine	562.2	222.9/277.9	20/20	35/25
α-ergocryptine	576.3	222.9/277.9	20/20	35/25
α-ergocryptinine	576.3	222.9/208.1	20/20	35/40
Ergotamine	582.2	222.9/208.1	20/20	35/40
Ergotaminine	582.2	222.9/208.1	20/20	35/40
Ergocristine	610.3	222.8/267.9	20/20	35/25
Ergocristinine	610.3	222.8/305.2	20/20	35/30

^1^ Precursor ion for each EAs was [M+H]^+^; ^2^ quantifier/qualifier.

**Table 2:** The percentage of positive samples, the mean values (calculated with a medium bound approach), the standard deviation and the maximum contamination level obtained for each of the analysed EAs are reported.

Wheat	Ergometrine/-inine	Ergosine/-inine	Ergocornine/-inine	α-ergocryptinine/-inine	Ergotamine/-inine	Ergocristine/-inine
Flour (*n* = 16)						
Positive (%)	50/38	56/-	56/-	44/-	6/-	6/-
Mean (µg/kg)	3.5/2.0	2.2/-	2.5/-	1.9/-	1.3/-	1.3/-
s_r_ (µg/kg)	2.5/1	0.9/-	1.2/-	0.9/-	0.3/-	0.3/-
Max (µg/kg)	8.1/3.9	3.2/-	5.0/-	4.2/-	2.6/-	2.6/-
Bread (*n* = 39)						
Positive (%)	36/26	28 /18	31/31	46/23	18/21	56/39
Mean (µg/kg)	7.7/2.2	10.5/2.1	7.6/2.8	5.9/3.1	12.7/2.9	11.2/6.7
s_r_ (µg/kg)	17.3/2.4	32.5 /3.2	17.1/3.9	9.6/6.5	50.8/7.1	28.4/18.6
Max (µg/kg)	89.8/14.8	191.8/20.5	81.3/23.5	39.9/40.7	314.3/45.2	168.1/116.2
**Rye**						
Flour (*n* = 4)						
Positive (%)	-	50/-	-	25/-	25/-	50/25
Mean (µg/kg)	-	5.0/-	-	2.1/-	3.3/-	7.9/2.6
s_r_ (µg/kg)	-	6.6/-	-	1.8/-	4.1/-	11.7/2.8
Max (µg/kg)	-	14.9/-	-	4.8/-	9.5/-	25.3/6.8
Bread (*n* = 12)						
Positive (%)	-/42	58/17	33/17	42/25	42/25	58/22
Mean (µg/kg)	-/6.4	11.0/1.6	2.3/1.7	2.6/2.1	3.8/1.8	9.1/5.0
s_r_ (µg/kg)	-/10.0	15.7/1.0	1.7/1.1	2.1/1.7	3.2/1.1	11/6.2
Max (µg/kg)	-/35.2	53.4/4.4	6.8/5.0	7.3/7.0	12/4.7	34.7/50

## Supplementary Materials

The following are available online at https://www.mdpi.com/2304-8158/8/5/150/s1, Table S1: Performance characteristics obtained during method verification for the selected EAs.
