# Supplementary material for: Ergot Alkaloids in Wheat and Rye Derived Products in Italy
Source: Foods. 2019 May 1;8(5):150. doi: 10.3390/foods8050150 (PMC6560453; doi:10.3390/foods8050150)
Supplement: Supplementary file 1 [file foods-08-00150-s001.pdf]

Table S1. Performance characteristics obtained during method verification for the selected EAs.

		Ergometrine/-inine	Ergosine/-inine	Ergocornine/-inine	α-ergocryptinine/-inine	Ergotamine/-inine	Ergocristine/-inine
**Wheat flour**							
R_A (%)	Mean value	124/118	98/110	101/86	84/111	97/98	87/87
	s	25/30	18/13	11/9	10/13	19/5	17/17
	RSD_r	20/25	18/12	11/10	12/12	20/5	19/19
SSE (%)	Mean value	153/149	102/129	123/74	85/131	108/123	104/114
R_E (%)	Mean value	81/79	96/85	82/117	99/85	90/80	84/76
**Wheat bread**							
R_A (%)	Mean value	120/67	89/115	102/115	111/135	128/114	86/78
	s_r	30/13	11/13	15/9	18/24	23/8	17/12
	RSD_r	25/20	12/11	15/8	16/18	18/7	20/16
SSE (%)	Mean value	136/91	109/137	109/134	150/159	135/148	92/124
R_E (%)	Mean value	88/74	82/84	94/86	74/85	94/77	93/63
**Rye flour**							
R_A (%)	Mean value	113/68	87/117	102/111	117/126	145/129	82/74
	s_r	15/8	8/14	13/11	16/19	17/15	17/11
	RSD_r	13/12	9/12	13/10	14/15	12/12	21/15
SSE (%)	Mean value	128/92	133/139	10/984	120/132	154/152	88/118
R_E (%)	Mean value	88/74	65/84	98/113	98/95	94/71	93/63
**Rye bread**							
R_A (%)	Mean value	100112	85/12	107/99	111/123	128/124	86/78
	s_r	16/28	7/28	13/12	10/32	8/20	17/20
	RSD_r	16/25	8/25	12/12	9/26	6/16	20/26
SSE (%)	Mean value	114/151	104/133	114/129	132/145	136/143	92/107
R_E (%)	Mean value	88/74	82/84	94/77	84/85	94/87	93/73
